# Unraveling the Health Impacts of Air Pollution: Assessments and Mechanisms

**DOI:** 10.3390/toxics14070624

**Published:** 2026-07-17

**Authors:** Jia Xu, Ting Wang, Tingting Ku

**Affiliations:** 1 State Key Laboratory of Environmental Criteria and Risk Assessment, Chinese Research Academy of Environmental Sciences, Beijing 100012, China; xu.jia@craes.org.cn; 2 College of Environmental Science and Engineering, Nankai University, Tianjin 300071, China; 3 School of Environment and Resource Sciences, Shanxi University, Taiyuan 030006, China; kutingting@sxu.edu.cn

## 1. Introduction

Air pollution has been widely recognized as a major environmental risk factor for human morbidity and mortality. The World Health Organization (WHO) has reported that almost the entire global population breathes air that exceeds recommended air quality limits, highlighting the continuing public health burden of ambient air pollution [1]. Although numerous epidemiological and toxicological studies have demonstrated associations between air pollutant exposure and adverse health outcomes, the mechanisms and pathways linking external exposure, internal dose, biological response, and disease development remain incompletely understood [2,3].

Accurate exposure assessment is essential for evaluating the health impacts of air pollution. This includes both ambient exposure assessment and the identification of biomarkers of exposure or internal exposure markers. In addition, because air pollution is a complex mixture of gases, particulate matter, and associated chemical components, it is critical to identify key toxic pollutants, chemical components, and sources that drive adverse health effects [4,5,6]. Further mechanistic studies are also needed to clarify the biological and molecular pathways through which air pollutants affect different organs and susceptible populations [7,8].

This Special Issue, entitled “Unraveling the Health Impacts of Air Pollution: Assessments and Mechanisms”, aims to present recent advances in exposure assessment, identification of key toxic pollutants and components, mechanisms of action, and innovative toxicity assessment methods. The eleven articles included in this Special Issue provide multidisciplinary evidence for understanding air pollution-related health risks from exposure to biological response.

## 2. An Overview of Published Articles

The eleven articles published in this Special Issue can be broadly categorized into four main research directions: external and internal exposure assessment of air pollutants and associated biomarkers; epidemiological evidence of air pollution-related health outcomes and susceptible populations; pollutant-specific and mixture-related toxicological effects; and molecular and cellular mechanisms underlying air pollution toxicity.

Firstly, several studies focused on exposure assessment and exposure markers. Zrieq et al. (Contribution 1) developed a deep learning framework for spatial–temporal modeling of air pollutants in Saudi Arabian cities. PM_10_, PM_2.5_, CO, and O_3_ were modeled using long short-term memory models, and the model performance was compared with random forest and XGBoost models. Balogh et al. (Contribution 2) investigated levoglucosan and its isomers as biomarkers of exposure to wood-burning smoke. Although levoglucosan is widely used as an atmospheric tracer of biomass combustion, the study found that urinary levoglucosan did not consistently reflect ambient exposure under real-world conditions. Collectively, these studies highlight the importance of improving exposure modeling methods and carefully validating exposure markers before using them as indicators of internal exposure.

Secondly, several studies provided epidemiological evidence linking air pollution exposure to diverse health outcomes and susceptible populations. Xie et al. (Contribution 3) conducted an updated meta-analysis on ambient air pollution and Alzheimer’s disease and Parkinson’s disease. Their findings suggested that ambient air pollution, particularly PM_2.5_, may contribute to increased risks of neurodegenerative diseases. Wang et al. (Contribution 4) examined the effect of ozone exposure on cardiovascular and cerebrovascular disease mortality among elderly residents in Shenyang, China, and reported a positive association between ozone exposure and mortality, as well as evidence of a joint effect between ozone and PM_2.5_. Liu et al. (Contribution 5) conducted a nationwide case–control study and found associations between regional air pollutant exposure and substandard semen quality. Collectively, these studies suggest that air pollution may affect multiple organ systems and that susceptibility differences should be carefully considered in health risk assessment.

Thirdly, this Special Issue includes studies and reviews addressing pollutant-specific toxicological effects. He et al. (Contribution 6) reviewed the synergistic toxicity of PM_2.5_ and ozone and summarized current evidence on their combined toxic effects and underlying mechanisms. The review highlighted that the combined effects of PM_2.5_ and ozone may differ from those of either pollutant alone and may be influenced by PM_2.5_ properties, exposure conditions, and target organs. Stauffer and Tat (Contribution 7) reviewed the toxic effects of sulfur dioxide, including its environmental sources, exposure routes, respiratory and extrapulmonary health effects, toxicity mechanisms, exposure assessment approaches, and remaining knowledge gaps. Yoo et al. (Contribution 8) examined the inhibitory effects of Aquadag, a black carbon surrogate, and their findings suggested that black carbon may induce cumulative biological stress through surface-mediated interactions. Collectively, these studies suggest that air pollution health research should move beyond single-pollutant concentration metrics and pay greater attention to pollutant mixtures, physicochemical properties, source characteristics, and pollutant-specific mechanisms.

Fourthly, several studies explored the biological and molecular mechanisms underlying air pollution toxicity. Panya et al. (Contribution 9) and Prommana et al. (Contribution 10) investigated the cellular responses of genetically distinct non-small-cell lung cancer (NSCLC) cell lines to PM_2.5_ exposure, including responses to acute PM_2.5_ exposure and biomass-haze PM_2.5_ exposure. Their findings suggested that PM_2.5_-induced biological responses may vary according to both particle source characteristics and the genetic background of exposed cells, with oxidative stress, transcriptomic remodeling, metabolic disturbance, immune modulation, and therapy resistance emerging as relevant pathways. Chen et al. (Contribution 11) further reviewed mitochondrial DNA-related mechanisms in ozone-induced inflammation, emphasizing the role of mitochondrial dysfunction, mitochondrial DNA release, innate immune activation, and inflammatory responses. Collectively, these studies provide mechanistic insights beyond conventional exposure–outcome associations, highlighting oxidative stress, mitochondrial dysfunction, inflammation, immune regulation, and transcriptional reprogramming as important pathways linking air pollution exposure to disease development and progression.

Overall, these articles collectively link exposure assessment, population-level health evidence, pollutant-specific toxicity, and mechanistic insights, providing a broad overview of current approaches to studying air pollution-related health risks.

## 3. Conclusions

The studies in this Special Issue provide an integrated understanding of air pollution-related health risks by connecting exposure assessment, epidemiological evidence, pollutant-specific toxicity, and molecular mechanisms. They indicate that the health effects of air pollution cannot be fully characterized by ambient concentration measurements alone, but require consideration of internal exposure markers or biomarkers, susceptible populations, pollutant mixtures, source characteristics, and biological responses. They further demonstrate that air pollution may affect multiple organ systems through complex pathways involving oxidative stress, mitochondrial dysfunction, inflammation, immune regulation, metabolic disturbance and transcriptional reprogramming. They also underscore the importance of validating exposure markers and incorporating population susceptibility into health risk assessment.

The contributions in this Special Issue highlight several important research gaps across the four major research themes. First, for external and internal exposure assessment, advanced spatiotemporal modeling approaches and exposure biomarkers require further validation. For example, single biomarkers such as urinary levoglucosan may not reliably represent biomass smoke exposure under real-world conditions, highlighting the need for multi-biomarker panels that can better link external exposure, internal dose, and source-specific exposure. Second, the epidemiological studies in this Special Issue suggest that evidence on air pollution-related health outcomes remains association-based and should be strengthened by designs and measurements that better support causal inference. Third, pollutant-specific and mixture-related toxicological effects remain insufficiently characterized, particularly regarding the combined toxicity of PM_2.5_ and ozone and the biological relevance of black carbon-related surface-mediated stress. Fourth, molecular and cellular mechanisms underlying air pollution toxicity require further validation beyond current cell-based and mechanistic evidence. For example, pathways related to oxidative stress, mitochondrial dysfunction, inflammation, and genotype-dependent responses should be tested in chronic, in vivo, and physiologically relevant models.

This Special Issue also highlights opportunities to translate findings from exposure assessment, epidemiology, toxicology, and molecular research into more targeted air pollution control and health protection strategies. Specifically, combined or multi-biomarker approaches may improve population exposure risk stratification by linking external exposure, internal dose, source-specific exposure, and susceptible populations. Mixture toxicity evidence may support the optimization of ambient air quality standards and coordinated pollution-control strategies beyond single-pollutant concentration limits. Moreover, mechanistic findings related to oxidative stress, mitochondrial dysfunction, and inflammation may inform the identification of early-warning biomarkers and potential targets for preventive interventions in vulnerable populations.

Overall, this Special Issue provides valuable multidisciplinary evidence for advancing accurate exposure assessment and mechanistic understanding of the health effects of air pollution. Collectively, these findings establish a foundation for future research integrating exposure science, epidemiology, toxicology, and molecular biology to improve health risk assessment and support evidence-based air quality management.

## Data Availability

No new data were created or analyzed in this study. Data sharing is not applicable to this article.
